# Low WIP1 Expression Accelerates Ovarian Aging by Promoting Follicular Atresia and Primordial Follicle Activation

**DOI:** 10.3390/cells11233920

**Published:** 2022-12-03

**Authors:** Su Zhou, Yueyue Xi, Yingying Chen, Fangfang Fu, Wei Yan, Milu Li, Yaling Wu, Aiyue Luo, Ya Li, Shixuan Wang

**Affiliations:** Department of Obstetrics and Gynecology, Tongji Hospital Affiliated to Tongji Medical College, Huazhong University of Science and Technology, 1095 Jiefang Ave, Wuhan 430030, China

**Keywords:** ovarian aging, WIP1, follicular atresia, apoptosis, primordial follicle activation

## Abstract

Our previous study demonstrated that ovarian wild-type P53-induced phosphatase 1 (WIP1) expression decreased with age. We hypothesized that WIP1 activity was related to ovarian aging. The role of WIP1 in regulating ovarian aging and its mechanisms remain to be elucidated. Adult female mice with or without WIP1 inhibitor (GSK2830371) treatment were divided into three groups (Veh, GSK-7.5, GSK-15) to evaluate the effect of WIP1 on ovarian endocrine and reproductive function and the ovarian reserve. In vitro follicle culture and primary granulosa cell culture were applied to explore the mechanisms of WIP1 in regulating follicular development. This study revealed that WIP1 expression in atretic follicle granulosa cells is significantly lower than that in healthy follicles. Inhibiting WIP1 phosphatase activity in mice induced irregular estrous cycles, caused fertility declines, and decreased the ovarian reserve through triggering excessive follicular atresia and primordial follicle activation. Primordial follicle depletion was accelerated via PI3K-AKT-rpS6 signaling pathway activation. In vitro follicle culture experiments revealed that inhibiting WIP1 activity impaired follicular development and oocyte quality. In vitro granulosa cell experiments further indicated that downregulating WIP1 expression promoted granulosa cell death via WIP1-p53-BAX signaling pathway-mediated apoptosis. These findings suggest that appropriate WIP1 expression is essential for healthy follicular development, and decreased WIP1 expression accelerates ovarian aging by promoting follicular atresia and primordial follicle activation.

## 1. Introduction

The age-related decline in ovarian function is a major challenge in women’s reproductive health. Ovarian aging, characterized by declined endocrine function and reproductivity, is closely associated with the quality and quantity of follicles. The decline in the ovarian follicular reserve is nonlinear and seems to accelerate with age [1,2]. The mechanisms of the accelerated depletion of follicles in later reproductive ages remain to be elucidated. Exploring the mechanism may help to develop targeted treatment strategies to slow down the depletion of ovarian follicles and delay ovarian aging. Through targeted intervention to delay ovarian aging, ovarian function decline or menopause-related diseases such as cardiovascular disease [3], osteoporosis [4], neurodegenerative diseases [5], etc., could also be improved.

Healthy follicular development and oocyte maturation are the prerequisites for endocrine function and obtaining high-quality oocytes. Follicular atresia is also a survival-of-the-fittest physiological phenomenon that occurs throughout the ovarian life. Abnormalities in follicular development and atresia will inevitably lead to abnormal ovarian function [6,7]. Both external and internal factors induce cell DNA damage. The DNA double-strand breaks can alter genetic integrity and cause deleterious damage to cells. Previous studies have demonstrated that decreased oocyte quality in older individuals is closely related to increased DNA damage [8]. DNA damage in granulosa cells also accumulates with age [9]. If the DNA damage cannot be repaired, cells undergo programmed cell death or senescence to avoid severe mutagenic consequences [10]. The size of the follicle pool and the depletion rate of primordial follicles determine the ovarian functional lifespan [11]. Follicle destiny is determined by the balance between proapoptotic and pro-survival molecules [7,11,12,13]. The molecular mechanisms that regulate follicular development and ovarian aging still need to be explored.

Wild-type P53-induced phosphatase 1 (WIP1), coded by *Ppm1d,* is a Ser/Thr protein phosphatase of PP2C family. A number of proteins, regulating the DNA damage response and cellular checkpoint pathways, could be dephosphorylated by Wip1 [14]. Previous studies suggested that Wip1 was highly expressed in various tumor tissues [15,16,17,18,19,20]. In recent years, increased attention has been paid to the regulatory role of WIP1 in aging [21,22,23]. The islet B cells and neural stem cells of aged mice exhibit lower expression of Wip1 compared with younger ones [24,25]. The lack of Wip1 phosphatase leads to overactivation of the p38MAPK pathway, promoting the enhanced expression of aging-related genes, such as INK4a and ARF, and accelerating aging [24]. Upregulation of WIP1 expression in mice using genetic engineering technology can increase the number of neural stem/progenitor cells and induce a higher degree of neuronal differentiation. In addition, augmenting WIP1 expression in aged animals can promote the formation of neurons and restore olfactory function [25]. Our recent study also indicated that the ovaries of aged mice exhibited significantly lower WIP1 expression than the younger ones [26]. However, whether WIP1 is involved in the ovarian aging process needs to be further explored.

Deletion of *Wip1* has been shown to cause abnormal spermatogenesis and sperm maturation in mice, resulting in declined fertility [27,28,29]. Our recent study has revealed that in neonatal mouse ovaries, WIP1 regulates primordial follicle development, indicating its potential role in regulating the ovarian reserve [26]. The regulatory role of WIP1 in follicular development and ovarian function remains to be further clarified. This study aimed to explore the specific role and mechanisms of WIP1 phosphatase in regulating ovarian follicular development in adult mice. Our objective was to investigate the possible roles of WIP1 and its related signaling pathways in ovaries to provide guidance for finding strategies to delay ovarian aging.

## 2. Materials and Methods

### 2.1. Mice

Postnatal day (PND) 14 and PND21 C57BL/6j female mice, purchased from the Center for Disease and Prevention, in Wuhan, Hubei, China, were sacrificed for primary granulosa cell or organ culture. The ovaries of immature female mice at postnatal day (PND) 14 contained a large number of small secondary follicles. Homogeneous secondary follicles can be obtained by acupuncture, which can be used for exploring the follicle growth in vitro. In the ovaries of immature female mice at postnatal day (PND) 21, secondary follicles developed into larger secondary follicles and small antral follicles, which contained more proliferative granulosa cells. Thus, the PDN21 mouse ovaries were used for primary granulosa cell culture. Thirty-six female mice for in vivo experiments (C57BL/6j, 6 weeks old, purchased from Beijing Huafukang Bio-Technology Co., Ltd., Beijing, China) were fed freely for one week to acclimate to the environment. All animals were fed with standard chow and water ad libitum under appropriate temperature and humidity. All the mice were killed by cervical vertebrae dislocation. All in vivo experiments were carried out in accordance with the approved guidelines by the ethics committee of Tongji Hospital Affiliated to Tongji Medical College, Huazhong University of Science and Technology.

### 2.2. Experimental Design

After one week of acclimation, the 7-week-old mice were divided into three groups (12 mice in each group): the vehicle group as the control group (Veh), and two WIP inhibitor (GSK2830371, HY-15832, MedChemExpress, Monmouth Junction, NJ, USA) treated groups: 7.5 mg kg^−1^ GSK2830371 group (GSK-7.5), 15 mg kg^−1^ GSK group (GSK-15). GSK2830371 is a selective Wip1 phosphatase inhibitor with oral activity [30]. Mice were respectively administered with vehicle or GSK2830371 twice per day (9:00 and 18:00) by oral gavage for 14 consecutive days. 

The following pure solvents (2% DMSO, 30% PEG300, 5% Tween 80, dd H_2_O) were added to the GSK2830371 in turn to prepare the GSK2830371 suspension with the concentrations of 1.25 and 2.5 mg/mL. ① The Veh group mice were treated with the vehicle solvent of the same volume contained 2% DMSO, 30% PEG300, 5% Tween 80, and dd H_2_O. ② Each mouse in GSK-7.5 group was treated with 1.25 mg/mL GSK2830371 with the volume of about 120–130 μL per dose (body weight: 20–22 mg) (daily total doses: 15 mg/kg). ③ Each mouse in GSK-15 group was treated with 2.5 mg/mL GSK2830371 with the volume of about 120–130 μL per dose (body weight: 20–22 mg) (daily total doses: 30 mg/kg).

Seven mice per group were sacrificed to extract blood samples and ovary samples within 2 h after the last administration (at the age of 9 weeks). All the left ovaries (7 samples per group) were fixed with 4% formaldehyde. The right ovaries (7 samples per group) were quick-frozen with liquid nitrogen and stored in a −80 °C refrigerator for subsequent Western blot experiments. The rest mice (5 mice per group) were used for subsequent estrous cycle monitoring and mating test for two rounds. 

### 2.3. Estrous Cycle Monitoring and Mating Test

After two weeks of treatment, the estrous cycles of the rest mice (5 mice per group) were monitored at the ages of 9 to 11 weeks. Vaginal smears were tested at 9:00 am every day for consecutive two weeks as previously described [31]. The stage of the estrous cycles was judged by the cytology under microscope. The first round mating test was carried out after two weeks of estrous cycle monitoring (at 11 weeks old). Two female mice were placed in a cage with a male mouse of the right age for 10 days. After 10 days, the male mice were moved away. The pregnancy status was determined by observing the vaginal pin and measuring the weight of the female mice every day. The pregnant cohabited female mice and their litter size were evaluated. The pups were moved away after birth. At the age of 16 weeks old, the second round of estrous cycle monitoring and mating test were carried out as before.

### 2.4. Immunohistochemistry

The collected mouse ovaries were embedded in paraffin and serially sectioned (5 μm). As previously described [31], the sections were incubated with primary antibody (anti-WIP1 (F-10) sc-376257, 1:200, Santa Cruz Biotechnology, Santa Cruz, CA, USA) overnight at 4 °C. The sections were incubated with a secondary antibody (HRP-labeled goat Anti-rabbit/mouse IgG (H + L), Servicebio, GB23204, 1:200) for 60 min at 37 °C on the next day. Then, the sections were visualized with DAB-HRP chromogenic agent (DAKO, K5007). The negative control was section incubated with mouse or rabbit IgG (H + L) (2 μg/mL, BA1046, BA1044, BOSTER, Wuhan, China). Microscopy and the images were obtained by microscope (version 1.8.1, Olympus, Tokyo, Janpan) with the cellSens Dimension software. Image Pro Plus software was used to evaluate the relative expression.

### 2.5. Serum AMH Level by ELISA

Blood samples of mice (7 mice per group) were collected after two weeks of GSK2830371 treatment. The serum was isolated from blood sampled by eyeball extirpating. Five serum samples from each group were qualified for AMH testing, setting 2 repeated samples. The enzyme-linked immune sorbent assay (ELISA) Kit (Bioss, Beijing, China) was used to detect the serum AMH levels according to the manufacturer’s instructions.

### 2.6. Follicle Counting

The ovaries after two weeks of GSK2830371 treatment were cut into pieces of 5 µm, and four sections were mounted on a glass slide. Briefly, every fourth slide with hematoxylin and eosin staining was observed under the microscope, and only follicles with oocytes were counted. Two persons counted independently, and the counting results were analyzed together [31].

### 2.7. Periodic Acid-Schiff (PAS) Staining

The ovary sections were deparaffinized and rehydrated. Then, the slides were placed in 0.5% periodic acid for 15 min and Schiff ’s reagent for 30 min. The slides were washed in dH_2_O three times between each step. Counterstaining with hematoxylin and the following steps were as routine procedures [32].

### 2.8. Apoptosis Test

The In Situ Cell Death Detection Kit (Roche, Basel, Switzerland) was applied for analyzing cell apoptosis. As the manufacturer’s instructions suggested, the ovarian sections were incubated with terminal deoxynucleotidyl transferase-mediated nick-end labeling (TUNEL) reaction mixture for 1 h at 37 °C. The images were acquired with the cellSens Dimension software and microscope (version 1.8.1, Olympus, Tokyo, Janpan). The TUNEL positive cells were calculated by Image-Pro Plus.

### 2.9. Western Blot

Total protein was isolated from mouse ovaries or cultured cells for Western blot experiments as described previously [31]. The primary antibody: Anti-WIP1 (F-10) (sc-376257, 1:200, Santa Cruz Biotechnology, CA, USA), Phospho-p53 (Ser15) (#9284, Cell Signaling Technology, Beverly, MA, USA), Bax (ab32503, Abcam, Cambridge, MA, USA), Bcl-2 (ab182858, Abcam, Cambridge, MA, USA), Cleaved-Caspase3 (ab49822, Abcam, Cambridge, MA, USA), WIP1 Polyclonal Antibody (A6204, ABclonal, Wuhan, China); anti-AKT (ab8805, Abcam, Cambridge, MA, USA), anti-phosphorylated (p) AKT1 (Ser473) (ab81283, Abcam, Cambridge, MA, USA), Anti-rpS6 (ribosomal protein S6) (A6058, ABclonal, Wuhan, China), Anti-phospho-rpS6 (S235/236, AP0296, ABclonal, Wuhan, China), Anti-p70S6 kinase (p70S6K) (A2190, ABclonal, Wuhan, China), Anti-p-p70S6K T421/S424 (AP0502, ABclonal, Wuhan, China), GAPDH (ANT012, AntGene), β-actin (ANT321, AntGene). Image Lab (Version 5.1, Java image processing software, BioRad, Hercules, CA, USA) was used to quantify the integrated light intensity. GAPDH expression was used to verify equal loading.

### 2.10. Follicle Isolation and Follicle Encapsulation

The ovaries of immature female mice at postnatal day (PND) 14 contained a large number of small secondary follicles. Homogeneous secondary follicles can be obtained by acupuncture, which can be used for exploring the follicle growth in vitro. The methods of follicle isolation and culture were described previously [33,34]. Briefly, the PND14 mouse ovaries were removed, and the follicles were mechanically dissected with syringe needles. Intact follicles (diameter: 120–140 μm) with a spherical oocyte in the center were selected for in vitro culture. The sodium alginate solution (Sigma-Aldrich, St. Louis, MO, USA. A2033; 2% (*w*/*v*)) and 50 mM CaCl_2_ + 140 mM NaCl solution were used to encapsulate follicles as previously described [33].

The follicle culture medium was α-minimal essential medium (Life technologies corporation, Gaithersburg, MD) supplemented with 10% FBS (10099; Gibco, Grand Island, NY, USA), 100 IU/mL of penicillin, 100 μg/mL of streptomycin, Insulin Transferrin Selenium (5 μg/mL of insulin, 5 μg/mL of transferrin, and 5 ng/mL of selenium) (Sigma-Aldrich, St. Louis, MO, USA) and 10 mIU/mL recombinant mouse follicle-stimulating hormone (rFSH: 8576-FS; R&D systems, Minneapolis, MN, USA). Follicles were cultured at 37 °C in 95% air-5% CO_2_ for 6 days. The cultured follicles were treated with different concentrations of WIP1 inhibitor (GSK2830371: 0, 0.1, 1.0, 5.0, 10.0 μM). The groups were defined as CON, or as 0.1, 1.0, 5.0, 10.0 μM GSK. The culture medium was changed every other day. Images of follicles were acquired every day with microscope (Olympus) with the cellSens Dimension software. Image J software (Version 1.48) was applied to measure the diameter of each follicle.

### 2.11. In Vitro Oocyte Maturation

After 6 days in culture, the remaining alginate around the follicles was degraded by alginate lyase at 37 °C for 20–25 min. For in vitro oocyte maturation (IVM), the cultured follicles were transferred to the IVM medium, consisting of αMEM, 1.5 IU/mL of hCG, 10% FCS, and 5 ng/mL EGF (epidermal growth factor), then placed in CO_2_ incubator for fifteen to sixteen hours. Then, the cumulus complex was incubated with hyaluronidase solution (0.1 mg/mL) to remove the cumulus granulosa cells with micropipette tip pipetting several times. The oocyte stages were defined as follows: germinal vesicle (GV) stage, the oocyte does not resume meiosis in response to hCG treatment, and the oocyte nucleus persists; germinal vesicle breakdown (GVBD), the nuclear membrane of oocytes disappear, but no polar body is observed; metaphase-II arrested oocyte (MII), the oocyte resumes meiosis, and a polar body can be observed under a dissecting microscope; degenerated, the oocyte is fragmented several pieces [34]. 

### 2.12. Immunofluorescence

Deparaffinized and rehydrated follicle sections were blocked with 10% goat serum for 60 min and then incubated with Ki67 antibody (1:200, GB111141, Servicebio, Wuhan, China) overnight at 4 °C. The sections were incubated with secondary antibodies (1:200, Alexa Fluo^®^488 Donkey anti Rabbit IgG (H + L), AntGene, ANT024) for 60 min at 37 °C in the next day. Rabbit IgG was used as the negative control. Images were acquired with microscope (Olympus) and the cellSens Dimension software. 

### 2.13. Primary Granulosa Cell Culture and siRNA Interference

The PDN21 mouse ovaries were used for primary granulosa cell culture. Primary granulosa cells were harvested and cultured as described in a previously published paper [35]. After 24 h in culture, the cells were transfected with the *Ppm1d*-siRNA (Si-*Ppm1d* group) and negative control siRNA (Si-Nc group) with lipofectamine 3000 reagent (Invitrogen, Carlsbad, CA, USA) according to the manufacturer’s instructions. Mouse *Ppm1d*-siRNA and control siRNA were purchased from Riobio, Inc. (Guangzhou, China). Mouse Ppm1d-siRNA: si-m-Ppm1d_003 (siG151016012651), the target gene sequence: GCACCGACGAAATGGCTTA. The morphology of granulosa cells was observed and recorded every day with microscope (Olympus) with the cellSens Dimension software. After transfection for 48 h, cells were harvest for subsequent experiments.

### 2.14. Cell Proliferation Detection

The primary granulosa cell proliferation was analyzed using the Cell-Light EdU Apollo643 In Vitro Kit (C10310-2, RiboBio, Guangzhou, China) according to instructions provided by the manufacturer. The cellSens Dimension and Image-Pro Plus software were used to acquire and analyze the images.

### 2.15. Cell Apoptosis Detection

The FITC-Annexin V Apoptosis Detection Kit I (BD Pharmingen™) was applied to detect cell apoptosis. The primary granulosa cells were treated with Si-RNA for 48 h. Then, harvested cells were incubated with Annexin V and PI for fifteen minutes. The cells labeled by FITC-Annexin V or PI were analyzed by flow cytometer (BD Pharmingen, San Diego, CA, USA). 

### 2.16. Statistical Analysis

Data are presented as means (SD) except where noted otherwise. Unpaired Student’s *t* tests, one-way ANOVA, Fisher’s exact test, or Chi-square test were applied for statistical comparisons. Statistical analysis was conducted using SPSS (version 13.0) and GraphPad Prism 8.0. Statistically significant difference was set at *p* < 0.05.

## 3. Results

### 3.1. WIP1 Expression in Follicles of Different Stages in Mouse Ovaries

**Figure 1:**
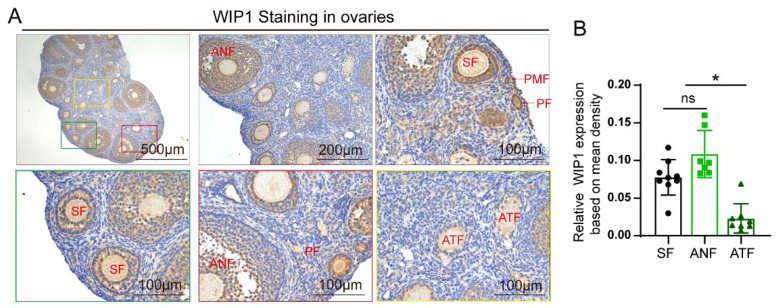
The expression of WIP1 in mouse ovaries. (**A**) Representative images of WIP1 expression in different stages of follicles in the mouse ovaries by IHC. (**B**) Relative WIP1 expression according to mean density (IOD/area). (* *p* < 0.05, one-way ANOVA) (PMF: primordial follicles, PF: primary follicles, SF: secondary follicles, ANF: antral follicles, THF: total healthy follicles, ATF: atretic follicles, CL: corpus luteum) Data are presented as the mean ± SD. * compared with the SF group. * *p* < 0.05.

The immunohistochemistry analysis in 6-week-old untreated wild-type mouse ovaries showed that WIP1 is mainly expressed in the granulosa cells and oocytes, and the stromal cells exhibited relatively weaker WIP1 expression. WIP1 was highly expressed in the granulosa cells of healthy follicles, while WIP1 expression was significantly downregulated in the granulosa cells of atretic follicles (Figure 1A). The relative WIP1 expression levels in follicles of healthy growing follicles (secondary follicles and antral follicles) and atretic follicles are shown in Figure 1B. The atretic follicles (ATF) showed significantly lower WIP1 expression compared with healthy growing follicles (secondary follicles (SF) and antral follicles (ANF)) (*p* < 0.05).

### 3.2. Inhibition of WIP1 Activity Induces the Irregular Estrus Cycles and Declined Reproductivity

**Figure 2:**
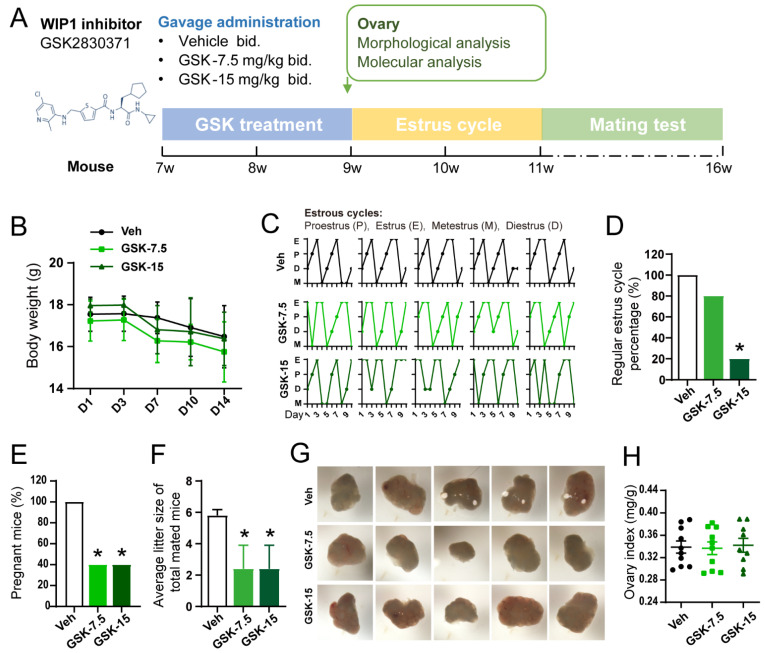
The effect of WIP1 inhibitor treatment on reproductive ability and estrous cycles. (**A**) Schematic diagram of the animal experiment. (**B**) Body weight during the gavage administration (*n* = 12, one-way ANOVA). (**C**) Representative estrous cycles of mice from Veh group, GSK-7.5 group and GSK-15 group. (**D**) The proportion of regular estrous cycles (*n* = 10, Fisher’s exact test). (**E**) The proportion of pregnant mice of total mated mice (*n* = 10, Fisher’s exact test). (**F**) Average litter size of total mated mice (*p* < 0.05, one-way ANOVA). (**G**) Representative ovary images. (**H**) Ovary index (ovary weight/body weight) (*n* = 10, one-way ANOVA). Black circle: Veh group; light green square: GSK-7.5 group; dark green triangle: GSK-15 group. Data are presented as the mean ± SD. * compared with the Veh group. * *p* < 0.05.

To confirm the role of WIP1 in ovarian function and its mechanism, the in vivo WIP1 inhibitor (GSK2830371) intervention experiment was carried out as shown in Figure 2A. The body weight of mice in the three groups showed no significant difference (Figure 2B). The estrus cycles were monitored after the GSK2830371 treatment. Representative estrus cycles are presented in Figure 2C. In the GSK-15 group, only 20% of mice showed regular estrous cycles, which was significantly lower than the Veh group (100%) (*p* < 0.05), while there was no significant difference between the GSK-7.5 group (80%) and the Veh group (100%) (Figure 2D). We observed that the pregnant rate in GSK-7.5 and GSK-15 groups (40%) was significantly lower than that of the Veh group (100%) (*p* < 0.05) (Figure 2E). The average litter size of total mated mice in the GSK-7.5 and GSK-15 groups (GSK-7.5: 2.40 ± 3.36; GSK-15: 2.40 ± 3.36) was lower than that of the Veh group (5.90 ± 0.84) (*p* < 0.05) (Figure 2F). Five representative ovary images are shown in Figure 2G, and ovary index (ratio of ovary weight to body weight) also showed no significant difference among the three groups (*p* > 0.05) (Figure 2H).

### 3.3. Inhibition of WIP1 Activity Leads to Declined Ovarian Reserve

**Figure 3:**
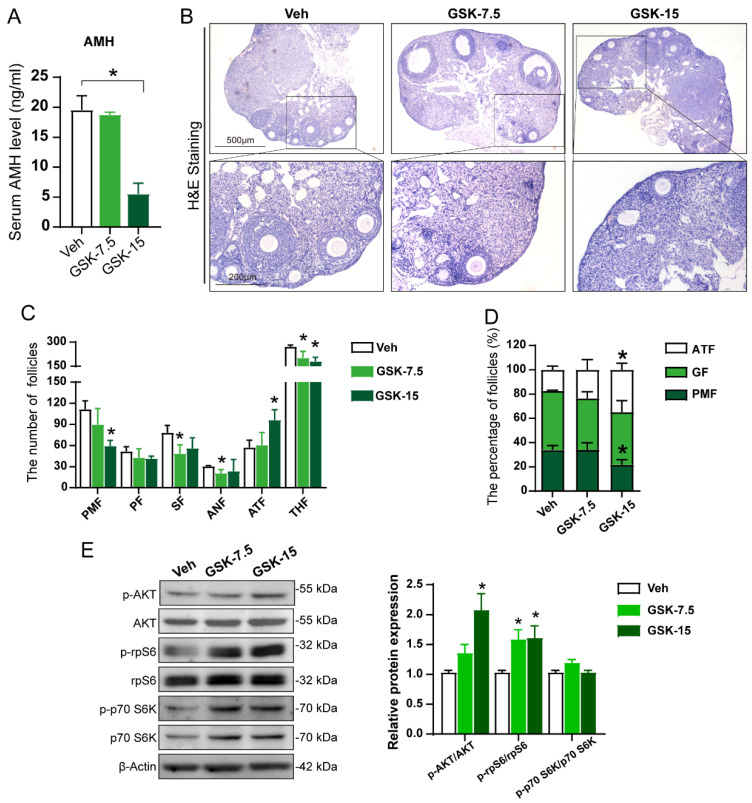
The WIP1 inhibitor treatment caused the decreased ovarian reserve in mice. (**A**) Serum AMH level (*n* = 5, one-way ANOVA). (**B**) The representative H&E staining images of mouse ovaries. (**C**) Follicle counting results according to ovary serial sections (*n* = 5, one-way ANOVA) (PMF: primordial follicles, PF: primary follicles, SF: secondary follicles, ANF: antral follicles, THF: total healthy follicles, ATF: atretic follicles). (**D**) The proportion of follicles at different stages (*n* = 5, one-way ANOVA). (**E**) The primordial follicle-activation-related AKT-rpS6 signaling pathway proteins detected by Western blot (*n* ≥ 3, one-way ANOVA). Data are presented as the mean ± SD. * compared with the Veh group. * *p* < 0.05.

The serum AMH level decreased significantly in the GSK-15 group (5.529 ± 4.03 ng/mL) compared with the Veh group, while the GSK-7.5 group (18.78 ± 0.84 ng/mL) showed no significant difference compared with the Veh group (19.55 ± 4.70 ng/mL) (Figure 3A). The serial ovarian sections were stained with H&E for follicle counting (Figure 3B). The GSK2830371-treated mice exhibited fewer healthy follicles and more atretic follicles. Compared with the Veh group (secondary follicles (SF): 78.33 ± 10.21, antral follicles (ANF): 30.33 ± 1.15), the number of secondary follicles and antral follicles in the GSK-7.5 group (SF: 48.50 ± 12.45, ANF: 20.50 ± 5.45) decreased significantly (* *p* < 0.05). In the GSK-15 group, the number of primordial follicles (PMF) decreased significantly (Veh: 111.67 ± 11.59, GSK-15: 59.33 ± 8.02), while the number of atretic follicles (ATF) significantly increased (Veh: 57.33 ± 10.26, GSK-15: 96.00 ± 14.93), when compared with the Veh group (* *p* < 0.05) (Figure 3C). Compared with the Veh group, the total healthy follicles in both GSK groups decreased significantly (Veh: 272.00 ± 10.14, GSK-7.5: 201.50 ± 41.49, GSK-15: 180.00 ± 26.91) (* *p* < 0.05). According to the follicle counting results, the proportion of primordial, growing (containing primary, secondary and antral follicles) and atretic follicles (Figure 3D) was calculated. The primordial follicle proportion decreased, and the atretic follicles increased significantly in the GSK-15 group compared with the Veh group (PMF: Veh: 33.91% ± 3.62%, GSK-15: 21.70% ± 4.23%; ATF: Veh: 17.40% ± 3.10%, GSK-15: 34.90% ± 5.45%) (* *p* < 0.05), and the percentage of growing follicles in the total number of follicles increased slightly with no significant difference (Figure 3D). According to the statistics of follicle counting, we found that inhibiting the activity of WIP1 phosphatase in mice could accelerate the depletion of the primordial follicle pool and the occurrence of follicular atresia.

Accelerated primordial follicle activation is the main reason for the decrease in primordial follicles. The PI3K-Akt-rpS6 signaling pathway, regulating primordial follicle activation, was detected by Western blot. The results indicated that the Akt-rpS6 signaling pathways are activated in the GSK groups compared with the Veh group (Figure 3E and Figure S1). These results suggest that inhibiting WIP1 phosphatase activity accelerated primordial follicle activation, decreasing the primordial follicle reserve.

### 3.4. Inhibition of WIP1 Activity Promotes Follicular Atresia through Apoptosis Pathway

**Figure 4:**
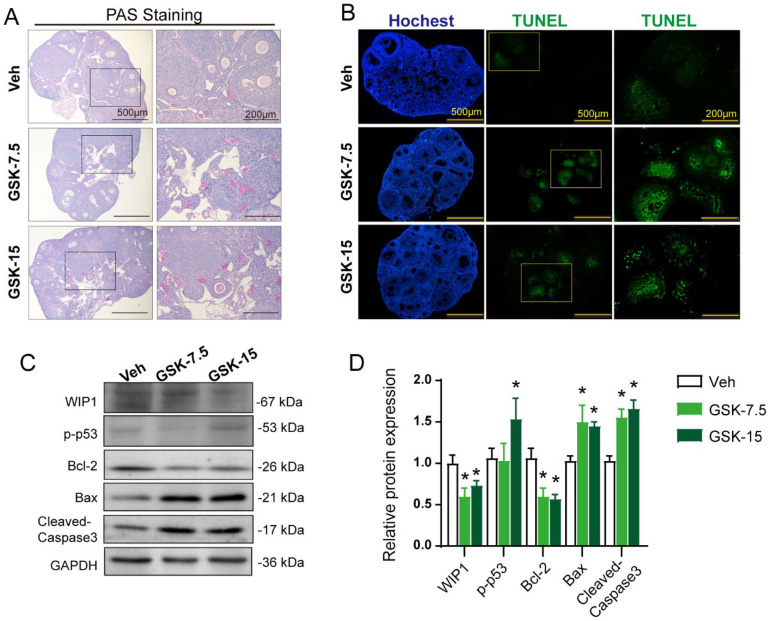
WIP1 inhibitor treatment promoted follicular atresia in mice. (**A**) PAS staining of mouse ovaries with or without GSK2830371 treatment. The ovaries from GSK2830371-treated mice exhibited more PAS positive staining. (**B**) TUNEL staining of mouse ovaries with or without GSK2830371 treatment. The ovaries from GSK2830371-treated mice exhibited more TUNEL positive staining. (**C**,**D**) Protein expression of apoptosis-related genes detected by Western blot (*n* ≥ 3, one-way ANOVA). Data are presented as the mean ± SD. *compared with the Veh group. * *p* < 0.05.

To accurately evaluate the status of the follicle in the ovary, we used PAS (periodic acid Schiff) staining. In ovarian tissue, PAS is positive in oocyte zona pellucida and follicular fluid of atretic follicles, while PAS is weak in healthy follicles [36]. PAS staining indicated that the GSK group had more PAS-positive staining sites and stronger staining than the Veh group (Figure 4A), suggesting that inhibiting WIP1 phosphatase activity could lead to more follicular atresia. Current studies have revealed that granulosa cell apoptosis is widely considered as the underlying mechanism of follicular atresia [7,12]. WIP1 has also been shown to be involved in regulating cell apoptosis [37,38]. The TUNEL results demonstrated that more apoptotic granulosa cells appeared in the GSK group ovaries than in the Veh group (Figure 4B). The key proteins involved in the apoptosis process were detected by Western blot (Figure 4C, Figure S2). The results showed a significant increase in p-p53 (S15), BAX and cleaved-Caspase3 expression in ovaries of GSK groups, when compared with the Veh group, while the anti-apoptosis protein Bcl-2 decreased significantly in the GSK groups (Figure 4D).

### 3.5. Inhibition of WIP1 Activity Impairs the Follicular Growth and Oocyte Quality In Vitro

**Figure 5:**
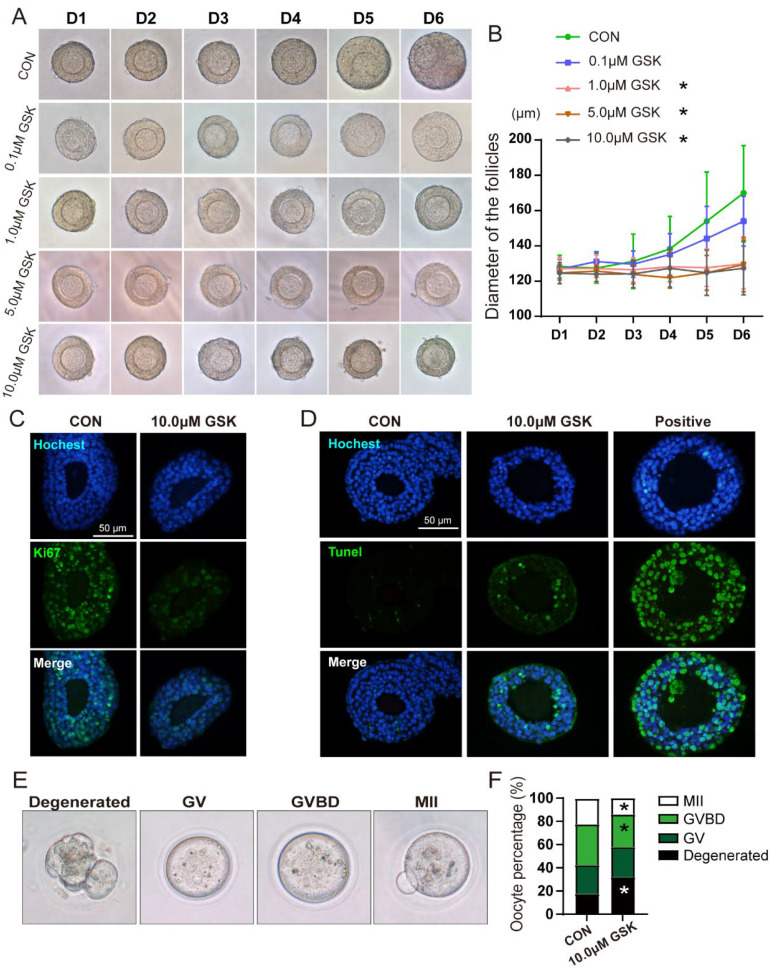
Effect of WIP1 inhibitor on secondary follicle development in vitro. (**A**) Morphological analysis of follicle growth. (**B**) Diameters of follicles treated with different concentrations of GSK2830371 (*n* ≥ 10, one-way ANOVA). (**C**) Ki67 staining of follicles cultured with GSK2830371 treatment. (**D**) Representative images of TUNEL staining of cultured follicles. (**E**) Microphotographs showing the in vitro maturation of follicular oocytes. (**F**) The percentages of oocytes in each stage were quantified (*n* ≥ 40, Chi-square test). (GV, germinal vesicle; GVBD, germinal vesicle breakdown; MII, metaphase II). Data are presented as the mean ± SD. * compared with the CON group. * *p* < 0.05.

To further clarify the influence of WIP1 on follicular development and atresia, preantral follicles were cultured with WIP1 inhibitor GSK2830371 (0, 0.1, 1.0, 5.0 and 10.0 μM) for six days. To explore the effect of WIP1 on the preantral follicle growth, the follicle morphology was observed and recorded daily (Figure 5A). The analysis of follicular diameter suggested that GSK2830371 intervention (1.0, 5.0 and 10.0 μM) could significantly inhibit follicular growth (Figure 5B). The follicles cultured without GSK2830371 or at 0.1 μM GSK2830371 kept growing and exhibited maximal volume at the 6th day of in vitro culture. The cell proliferation was detected by Ki67 staining, and follicles in the GSK group showed a reduction in Ki67 fluorescence intensity (Figure 5C). The TUNEL assay was applied to detect apoptosis in follicles after 4 days in culture. The TUNEL-positive granulosa cells in the GSK group were significantly increased compared with the CON group (Figure 5D). 

After 6 days in culture, the oocyte nuclear maturity was assessed to reflect the oocyte quality (Figure 5E). As shown in Figure 5F, compared with the CON group, the proportion of degenerated oocytes significantly increased in the GSK group (CON: 17.5%, GSK: 32.0%), and the oocytes in GVBD and MII phase declined (GVBD: CON 35.0%, GSK 28.0%; MII: CON 22.5%, GSK 14.0%) (*p* < 0.05). The results indicate that inhibition of phosphatase activity can limit follicular growth and oocyte maturation, through regulating proliferation and apoptosis of granulosa cells.

### 3.6. Downregulating WIP1 Promotes Mouse Granulosa Cell Apoptosis

**Figure 6:**
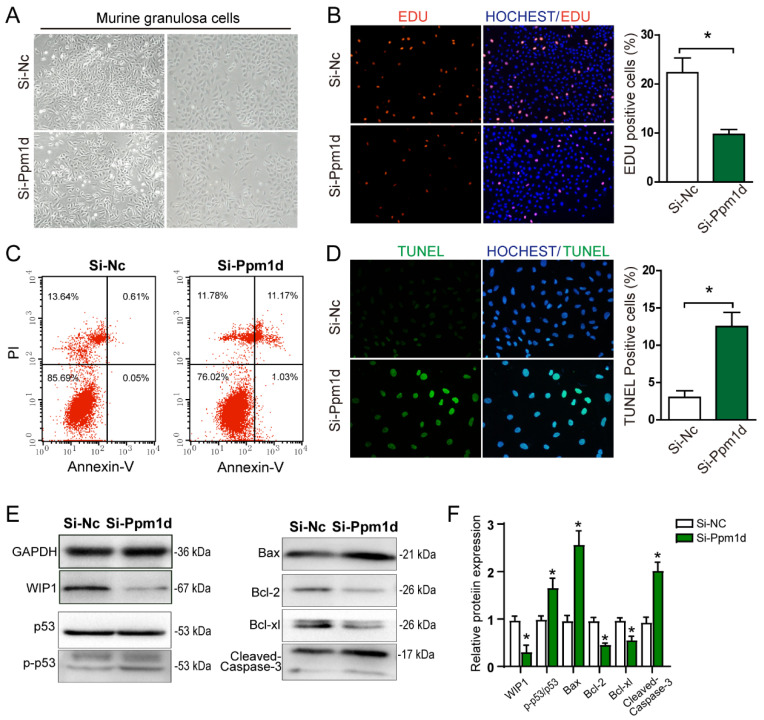
The effect of WIP1 on primary granulosa cells cultured in vitro. (**A**) The morphology of mouse granulosa cells. (**B**) Granulosa cell proliferation detected by EdU and the EdU-positive cell proportion. (**C**) Granulosa cell apoptosis detected by FACS. (**D**) Granulosa cell apoptosis detected by TUNEL and the TUNEL-positive cell proportion. (**E**,**F**) Protein expression of apoptosis-related genes detected by Western blot. Data are presented as the mean ± SD. * compared with the Si-Nc group. * *p* < 0.05.

Follicular development is closely related to granulosa cell viability. To verify the relationship between WIP1 expression and granulosa cell proliferation and apoptosis, primary mouse granulosa cells were isolated and cultured for 24 h and then transfected with Si-ppm1d-RNA. The representative images of granulosa cells in the Si-Nc group and the Si-Ppm1d group are shown in Figure 6A. After transfection for 48 h, EdU immunofluorescent staining results showed that cell proliferation decreased in the *Si-Ppm1d* group, in comparison with the Si-Nc group (Figure 6B). Cell apoptosis increased significantly in the *Si-Ppm1d* group, as evidenced by fluorescence-activated cell sorting (FACS) apoptosis detection (Figure 6C) and TUNEL staining (Figure 6D). Western blot results indicate that the p53 and mitochondrial apoptosis pathway was activated after downregulating WIP1 (Figure 6E,F, Figure S3). 

## 4. Discussion

This present study demonstrates that WIP1 expression of atretic follicles is significantly lower than that of healthy follicles. In vivo experiments indicated that inhibiting WIP1 phosphatase activity in mice induced irregular estrous cycles, caused fertility declines, and decreased the ovarian reserve through triggering excessive follicular atresia and primordial follicle activation. In vitro experiments suggest that WIP1-related abnormal apoptosis in granulosa cells leads to follicular atresia, also impairing the oocyte quality. WIP1 plays an essential role in regulating the ovarian reserve and ovarian function.

In our current study, WIP1 expression was demonstrated to be associated with follicular development. Atretic follicles exhibited lower WIP1 expression compared with the healthy ones. Follicular development and atresia are the key factors affecting ovarian function and ovarian life span. Follicular development and follicular atresia are finely regulated by a network of intracellular and extracellular signaling molecules [12]. The precise regulation of protein phosphorylation and dephosphorylation plays a key role in controlling various signaling pathways [39]. The phosphatase activity plays an essential role in the regulation of follicular development. PTEN, as a phosphatase, is shown to be necessary in the maintenance of the primordial follicle pool. PTEN phosphatase acts as the upstream of the PI3K-AKT pathway and helps to maintain the quiescence of primordial follicles. Deletion of PTEN causes overactivation of primordial follicles [40]. Our present study verified that WIP1 phosphatase also participates in the regulation of follicular development. 

WIP1, as a phosphatase, regulates the DNA damage response, apoptosis [41,42,43]. Recently, more studies focus on its role in the aging process [21,22,23], except for its role in tumorigenesis. One important hallmark of aging is the accumulation of DNA damage [44]. The endogenous and exogenous factors continually influence the stability and integrity of DNA [45]. A sophisticated network of DNA repair mechanisms has evolved in organisms. Emerging research indicates the importance of WIP1 in the regulation of stress-induced DNA damage and the relevant networks [41,46,47,48]. Our recently published data have confirmed that the expression of WIP1 in mouse ovaries decreased with age [26], which is consistent with the trends in other tissues [21,24,25]. The DNA damage accumulation and the decreased ability of DNA damage repair lead to more apoptosis in ovarian cells and follicle atresia. BRCA proteins (BRCA1, BRCA2) participated in the transcriptional regulation of DNA damage as well as the DNA repair process [49]. Clinical data and animal experiments have indicated that defects in BRCA1-related DNA double-strand break repair could induce ovarian reserve decline, accelerating ovarian aging [50]. The *Brca2*-deficient mice showed abnormal follicular development and oocyte quality impairment, because cellular DNA damage accumulated [51].

With increasing age, the ovarian reserve of mice decreased. The serum levels of anti-Müllerian hormone (AMH), which is produced by granulosa cells of small follicles, could well reflect the ovarian reserve [52]. Our previous study also suggested that WIP1 expression decreased with the increase in age [26]. In this current study, inhibiting WIP1 in young mice resulted in decreased serum AMH levels, which further confirmed the correlation between WIP1 expression and ovarian reserve in mice. Abnormal follicular development is the key to the decrease in ovarian reserve and ovarian function. Abnormal follicular development caused endocrine dysfunction eventually resulting in the irregularity of estrus cycles. Endocrine disorders (Figure 2C,D), follicular maturation disorders and decreased oocyte quality (Figure 5) were closely related to declined fertility. The litter size of pregnant mice showed no significant difference among groups. The average litter size of total mated mice decreased due to the decreased pregnancy rate in the GSK-7.5 and GSK-15 groups compared with the Veh group.

Low WIP1 activity induced excessive primordial follicle activation and follicular atresia, leading to decreased ovarian reserve. Previous studies have demonstrated that apoptosis is the main mechanism mediating follicular atresia [7,12]. Our data extend the previously identified role of apoptosis in follicular development and emphasize that the process is also regulated by WIP1. Some examples of WIP1 targets include p53, ATM and γ-H2AX, involving in cell cycle arrest, apoptosis, DNA repair, etc. [42]. Impaired DNA repair and cell survival could be induced by persistent ATM signaling activation. WIP1 could dampen the stress response through dephosphorylating p53 or ATM [53]. A recent study demonstrated that the WIP1-related p53 pathway controls cell fate through regulating apoptosis [54]. Consistent with our previous study [26], the current study demonstrated that inhibiting WIP1 activity led to enhanced apoptosis in ovarian granulosa cells, which eventually leads to follicular atresia and decreased ovarian reserve. This study also demonstrated that inhibiting WIP1 phosphatase in adult mice caused excessive primordial follicle depletion via activating the PI3K-AKT-rpS6 signaling pathway. Our recent published study has revealed that in neonatal mouse ovaries, inhibiting WIP1 phosphatase accelerates primordial follicle atresia and does not significantly activate the primordial follicle activation-related PI3K-AKT-rpS6 signaling pathway [26]. These results indicate that WIP1 is involved in the regulation of follicular development, while there are some differences in neonatal mouse ovaries and adult mouse ovaries.

The in vitro follicle culture experiments further demonstrated that inhibiting WIP1 phosphatase promoted granulosa cell apoptosis and follicular atresia and led to impaired follicular maturation and decreased oocyte quality. A previous study reported that WIP1 suppresses DNA repair in oocytes [55]. Inhibition of WIP1 phosphatase could improve the ability of oocytes to repair DNA damage [55]. Our current study mainly explored the role of WIP1 in granulosa cells. The role of WIP1 in granulosa cells seems different from its role in oocytes. Our study indicated that inhibition of WIP1 phosphatase may impair DNA damage repair ability and promote granulosa cell apoptosis through p53 signaling pathway, resulting in more follicular atresia and declined ovarian reserve. Previous studies have demonstrated that *Wip1* deficiency affects spermatogenesis and sperm maturation, resulting in reduced fertility in males [27,28,29]. Similar to the studies in male reproductivity, our study also indicates the low WIP1 activity associated with poor ovarian function.

Our previous study has already demonstrated that WIP1 expression in mouse ovaries declined with age [26]. The WIP1 phosphatase activity decline is closely associated with impaired follicular development, as well as ovarian aging. The age-dependent WIP1 downregulation accelerated the atresia of growing follicles, directly or indirectly accelerating depletion of the primordial follicles. Consistent with our study, WIP1 has been proven to play a key role in several physiological processes such as neurogenesis and organismal aging [24,25]. Another study also shows that WIP1 is highly expressed in hematopoietic stem cells (HSC) but decreases with age, and *Wip1*-deficient mice exhibiting multifaceted HSC aging phenotypes, depending on the distinct effects of p53 and mTORC1 pathways governed by WIP1 [56]. WIP1 also participates in preventing aging-related B-cell development dysfunction [57]. Collectively, low WIP1 expression or WIP1 deficiency is related to organismal aging, which is consistent with the results in the current study. In this study, we only demonstrated that low expression of WIP1 is detrimental to ovarian function. In the future, we still need to demonstrate whether augmenting Wip1 expression by genomic or pharmacologic methods in aged animals could improve the ovarian reserve and delay ovarian aging.

## 5. Conclusions

In summary, the current study reveals that inhibition of WIP1 activity accelerates follicular atresia via the WIP1-p53-mediated mitochondrial apoptosis signaling pathway in granulosa cells. Meanwhile, primordial follicle depletion is accelerated via PI3K-AKT-rpS6 signal activation. Appropriate WIP1 expression is essential for healthy follicular development and oocyte quality, and decreased WIP1 expression accelerates ovarian aging by promoting follicular atresia and primordial follicle activation.

## Data Availability

The original data that support the findings of this study are included in the article; further inquiries can be directed to the corresponding author.

## Supplementary Materials

The following are available online at https://www.mdpi.com/article/10.3390/cells11233920/s1, Figure S1: The original images of the Western blot experiments for Figure 3E. Figure S2: The original images of the Western blot experiments for Figure 4C. Figure S3: The original images of the Western blot experiments for Figure 6E.
