# Supplementary material for: Low WIP1 Expression Accelerates Ovarian Aging by Promoting Follicular Atresia and Primordial Follicle Activation
Source: Cells. 2022 Dec 3;11(23):3920. doi: 10.3390/cells11233920 (PMC9736686; doi:10.3390/cells11233920)

Supplementary materials

Figure S1

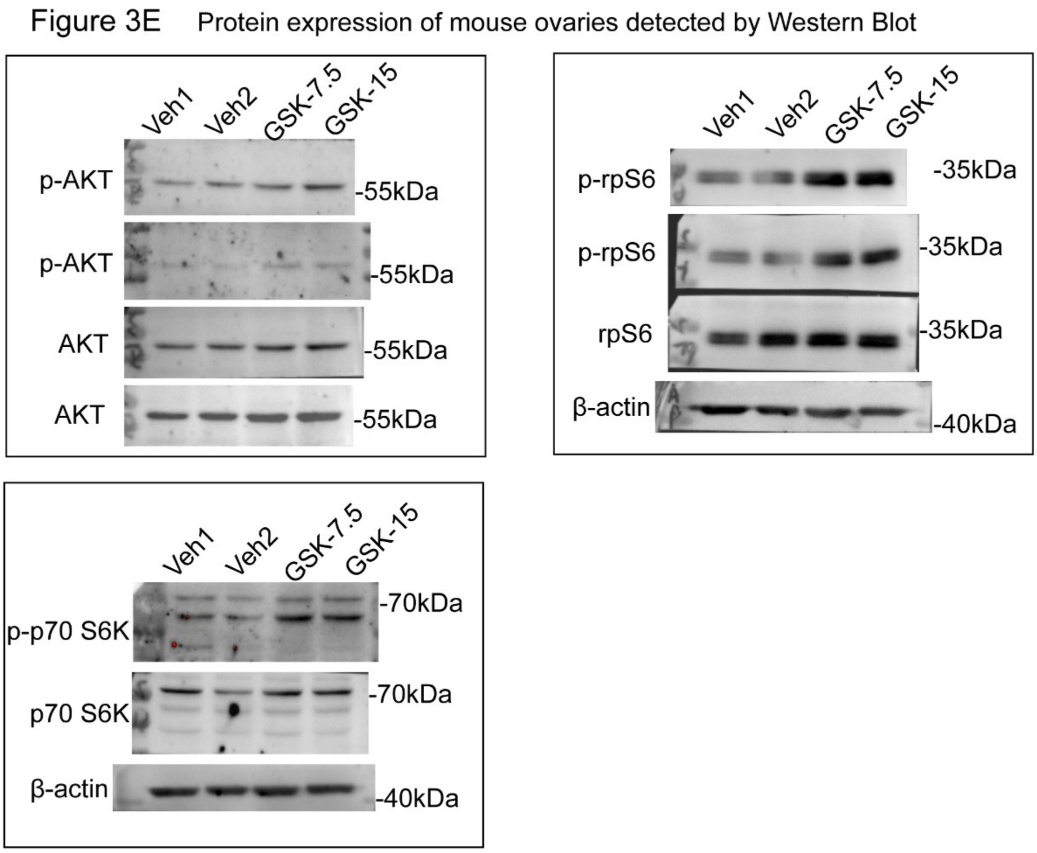

Figure S2

Figure 4C Protein expression of mouse ovaries detected by Western Blot

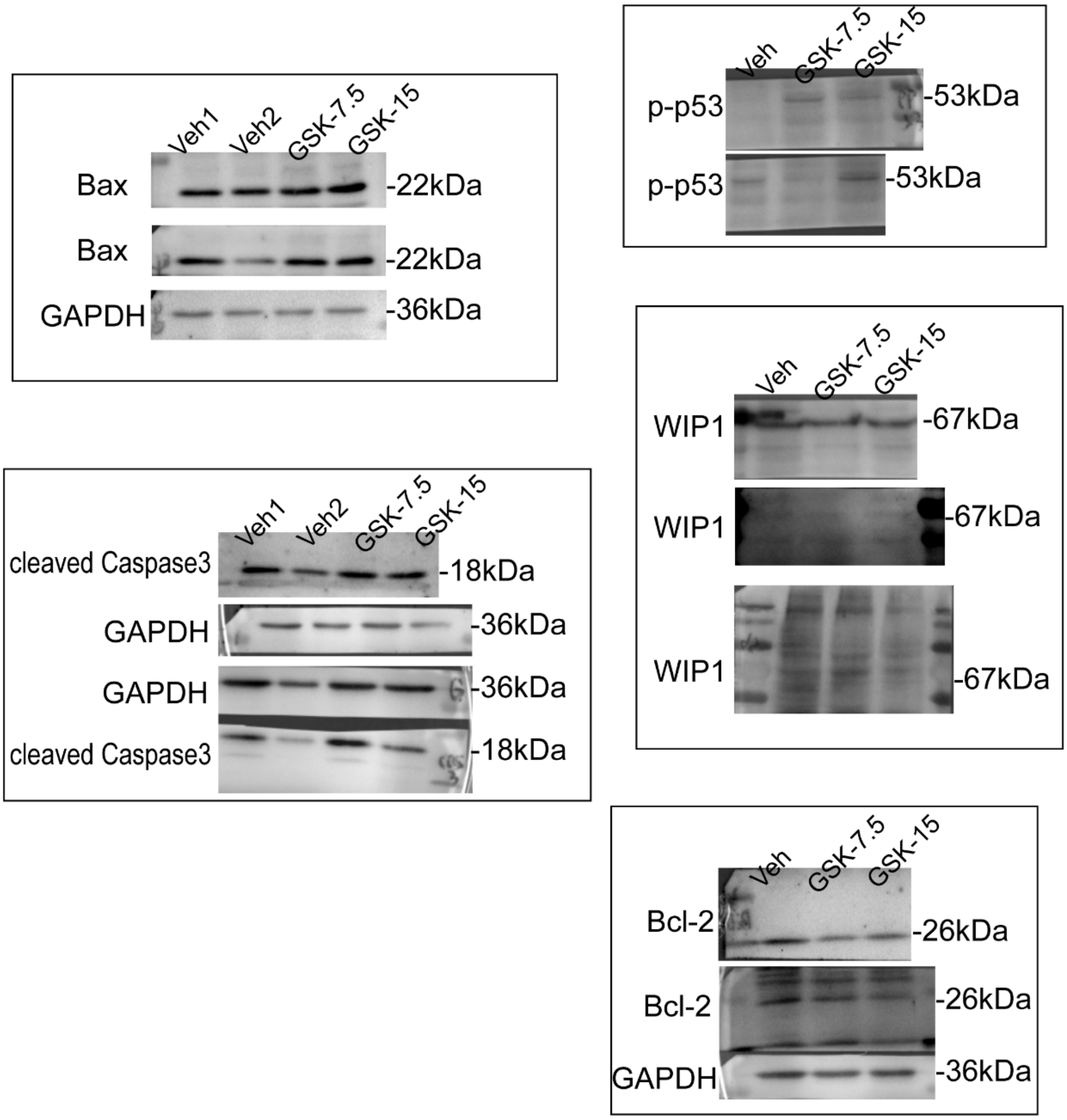

Figure 4C Protein expression of mouse ovaries detected by Western Blot

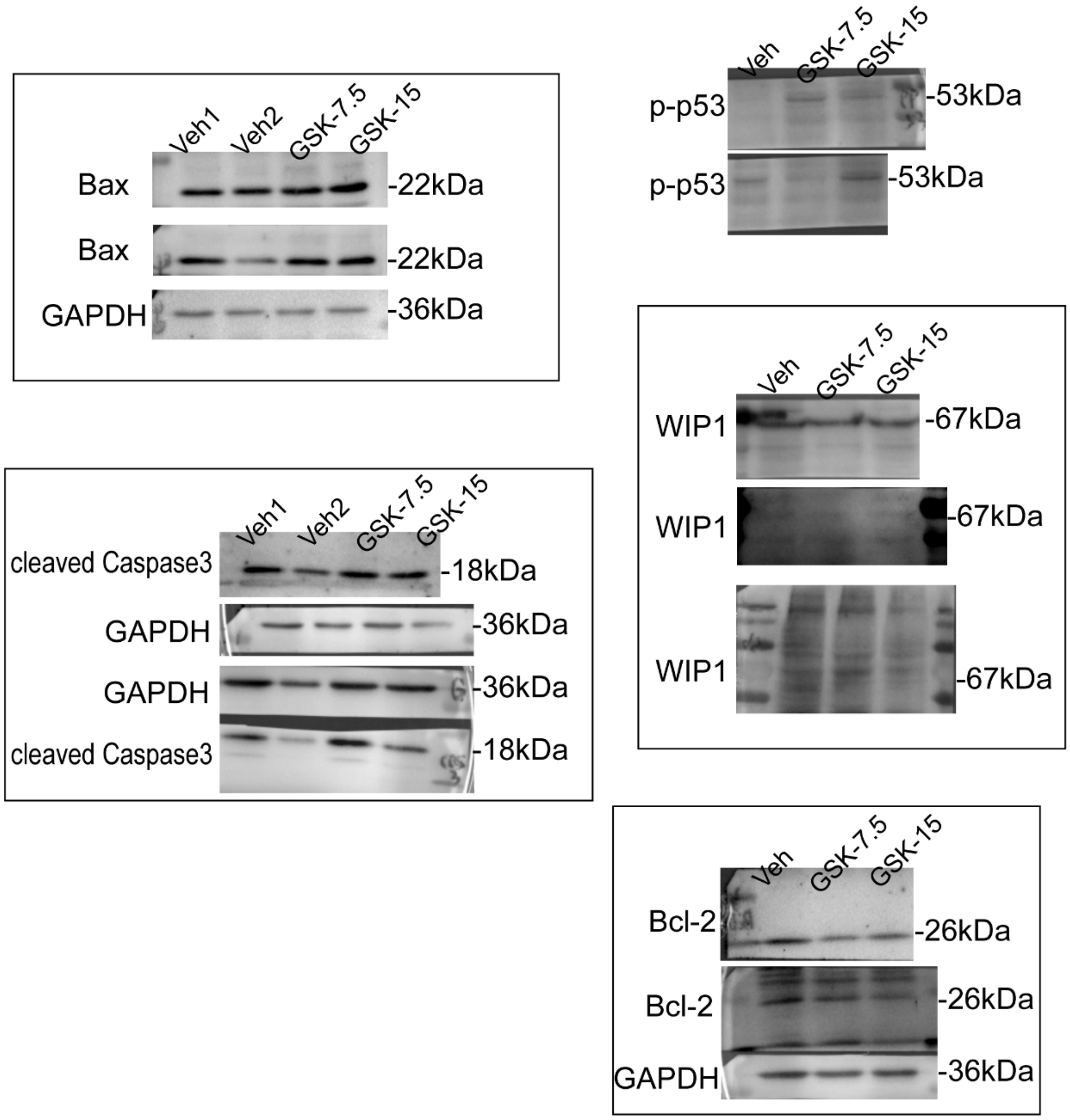

Figure S3

Figure 6E Protein expression of granulosa cells detected by Western Blot

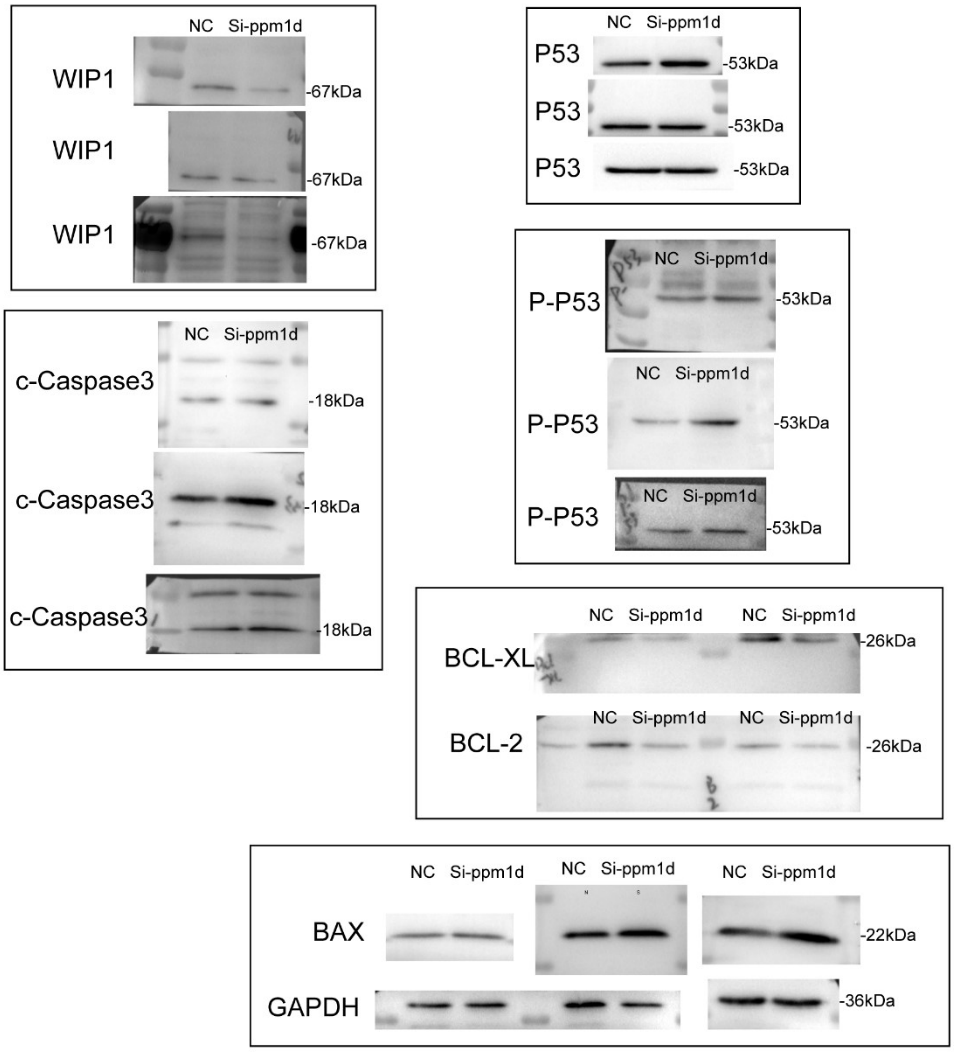

Supplement: Supplementary file 1 [file cells-11-03920-s001.zip › cells-2019830-supplementary.pdf]
